# Supplementary material for: Change detection in the primate auditory cortex through feedback of prediction error signals
Source: Nat Commun. 2023 Nov 13;14:6981. doi: 10.1038/s41467-023-42553-3 (PMC10643402; doi:10.1038/s41467-023-42553-3)
Supplement: Supplementary file 1 — Supplementary Information [file 41467_2023_42553_MOESM1_ESM.pdf]

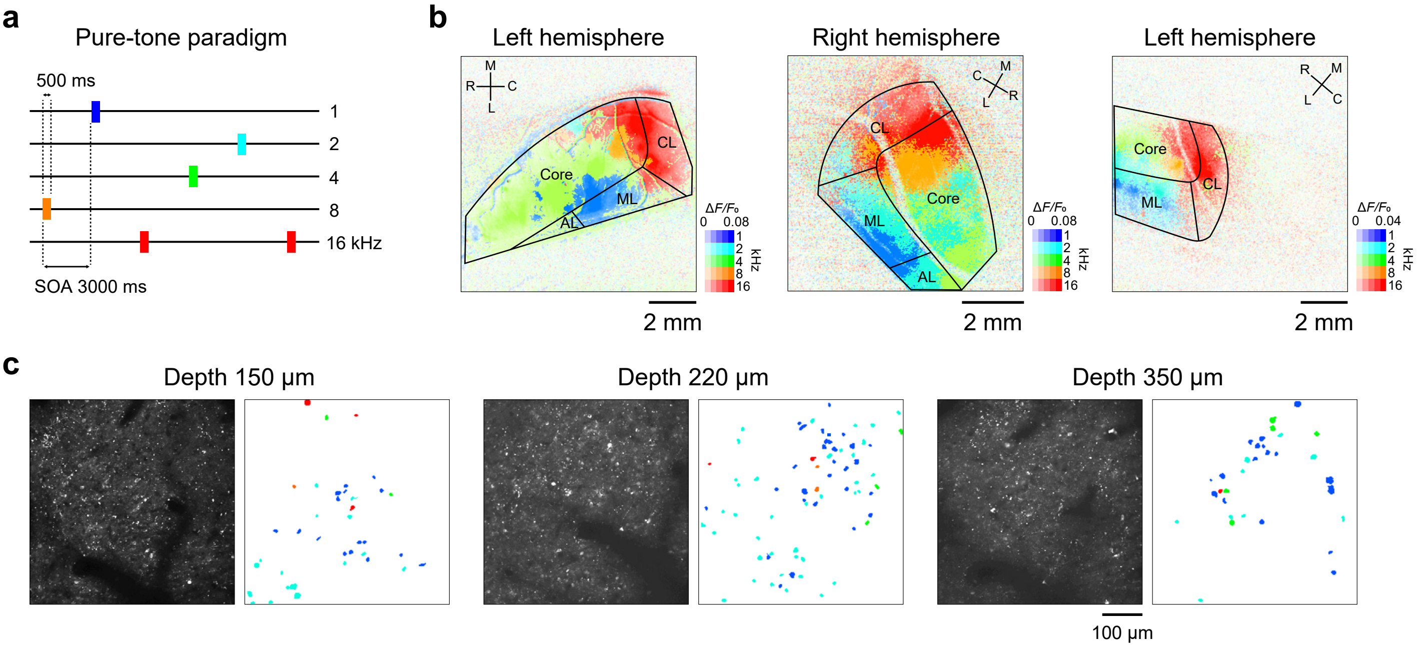

Supplementary Fig. 1. Additional tonotopic maps and responses of individual neurons in the core. **a**, Time sequence of the pure-tone paradigm. The tone duration was 500 ms. **b**, Best frequency maps from three other awake animals. Black lines indicate the outer lines of imaging windows or putative contours of the sub-regions of the auditory cortex. **c**, Representative two-photon images of the same field of view at three different depths from the cortical surface and tone-responsive neurons in the corresponding planes. The color in each neuron indicates the assigned BF.

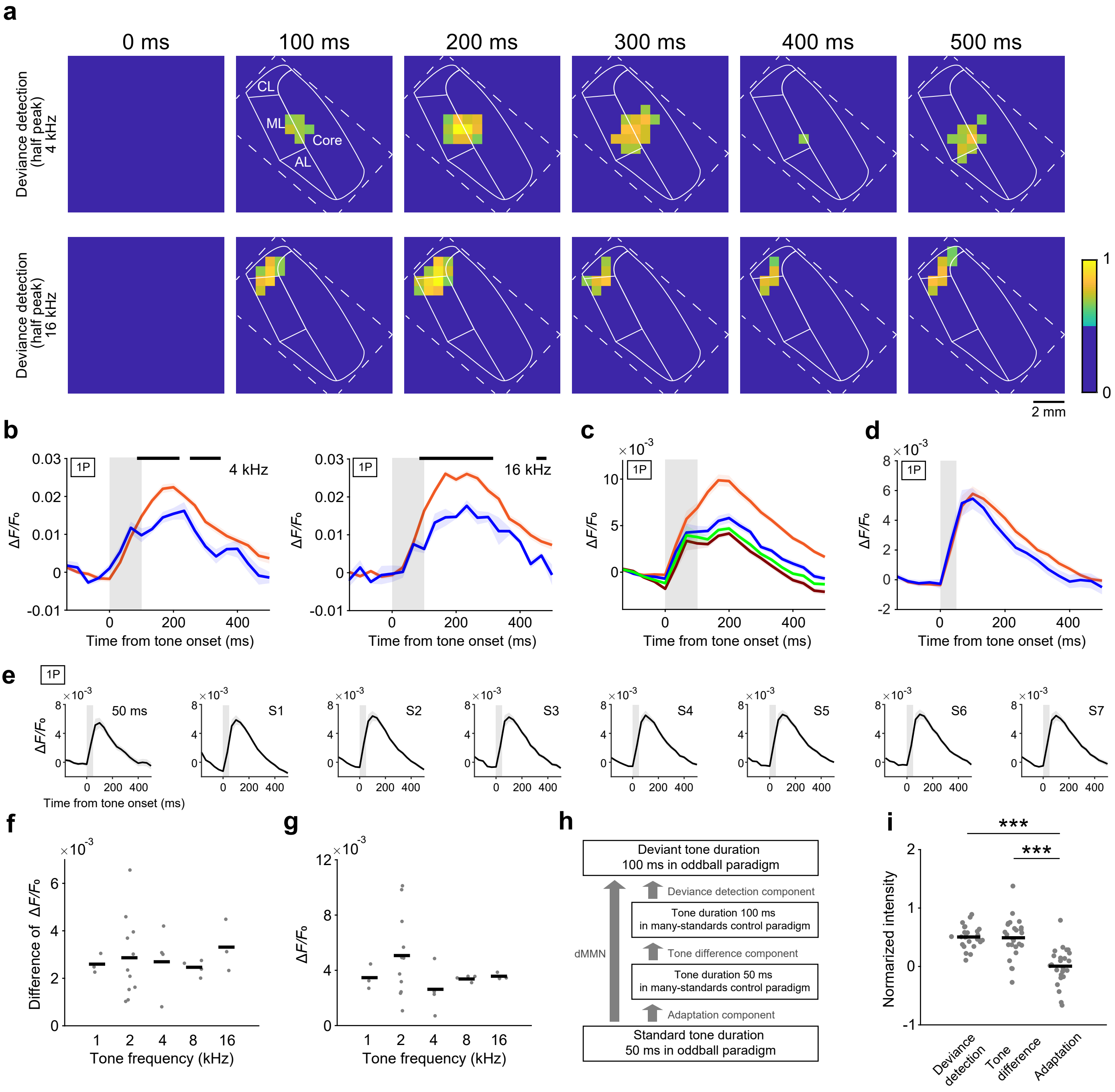
a
0 ms
100 ms
200 ms
300 ms
400 ms
500 ms
Deviance detection (half peak) 4 kHz
CL
ML
Core
AL
Deviance detection (half peak) 16 kHz
1
0
2 mm
b
1P
4 kHz
16 kHz
ΔF/F₀
Time from tone onset (ms)
c
d
e
50 ms
S1
S2
S3
S4
S5
S6
S7
f
Difference of ΔF/F₀
Tone frequency (kHz)
g
h
Deviant tone duration
100 ms in oddball paradigm
Deviance detection component
Tone duration 100 ms
in many-standards control paradigm
dMMN
Tone difference component
Tone duration 50 ms
in many-standards control paradigm
Adaptation component
Standard tone duration
50 ms in oddball paradigm
i
Normarized intensity
***
***
Deviance detection
Tone difference
Adaptation

Supplementary Fig. 2. Additional one-photon calcium responses of the core area in the dMMN paradigm. a, Time course of the deviance detection map in the field of view shown in Fig. 2c. The tone frequency was 4 kHz (top) and 16 kHz (bottom). **b**, Two representative time courses of calcium responses shown in **a** (left and right). Orange, calcium responses to the deviant 4 kHz (left) and 16 kHz (right) tones (100 ms, n = 28 trials for left and 29 trials for right). Blue, calcium responses to the 100 ms corresponding tone in the many-standards control paradigm (n = 8 trials for left and 4 trials for right). Black lines indicate the timepoints at which the response amplitude was significantly different between these paradigms (P < 0.05 Wilcoxon rank sum test, two-sided, FDR-adjusted). Shading on each line indicates SEM. **c**, Averaged time course of calcium responses for the dMMN paradigm. The responses were averaged irrespective of the tone frequency. Orange and blue traces are identical to those in Fig. 2e. A brown trace indicates calcium responses to a 100 ms tone whose previous tone had ≥ 75 ms duration in the many-standards control paradigm, and a green trace indicates the average response to all 100 ms tones in the many-standards control paradigm. Brown and green traces represent responses subject to the large decay of calcium responses in the previous tone stimulus and the fluorescent signal averaged for 150 ms before the tone onset being set to zero; these traces for 100 ms before the tone onset were slightly different from those of the orange and blue traces. However, the large response to the deviant tone was apparent compared with the other three responses (see Methods; n = 25 dMMN paradigms). Shading on each line indicates SEM. **d**, Averaged time course of calcium responses to the 50 ms tone in the oddball paradigm (orange) and the 50 ms tone in the many-standards control paradigm (blue). In the former responses, only the tone stimulus immediately before the deviant tone stimulus was chosen (n = 25 dMMN paradigms). The averaged amplitude during 0–200 ms after the tone onset was not significantly different (P = 0.14, paired t-test, two-sided). Shading on each line indicates SEM. **e**, Top left, averaged time course of calcium responses of the core regions to the 50 ms tone in the many-standards control paradigm (n = 25 dMMN paradigms from three animals). In the other panels, the averaged time courses of calcium responses of the core regions to the ith 50 ms tone (labeled by Si, i = 1, 2, to 7) after the deviant tone stimulus in the oddball paradigm are shown. The adaptation to the repetitive 50-ms stimuli was not apparent. Shading on each line indicates SEM. **f**, Mean amplitude of the deviance detection in each tone frequency used for the oddball paradigm (averaged over 200–400 ms after the tone onset) of one-photon responses in the core. There was no significant difference among the frequencies (Black bars indicate the mean. P > 0.05, Wilcoxon rank sum test with Bonferroni correction, two-sided, n = 25; 3 for 1 kHz, 11 for 2 kHz, 4 for 4 kHz, 4 for 8 kHz, and 3 for 16 kHz, from three animals). **g**, Mean amplitude (averaged over 0–200 ms after the tone onset) of the core one-photon response to the 100-ms tone in the many-standards control paradigm with each tone frequency. There was no significant difference among the five frequencies (Black bars indicate the mean. P > 0.05, Wilcoxon rank sum test with Bonferroni correction, two-sided, n = 25, the same as in **f**). **h**, dMMN consisting of the deviance detection, tone difference, and adaptation components. **i**, Contributions of the three components in the core. The data are the same as those in Fig. 2e (core, n = 25 sessions from three animals). For each session, each component amplitude (for 200–400 ms after the tone onset) was normalized to the dMMN amplitude. Paired t-test with Bonferroni correction, two-sided. *** $P = 1.2 \times 10^{-6}$, d = 1.94 for Deviance detection vs. Adaptation, *** $P = 9.0 \times 10^{-4}$, d = 1.49 for Tone difference vs. Adaptation.

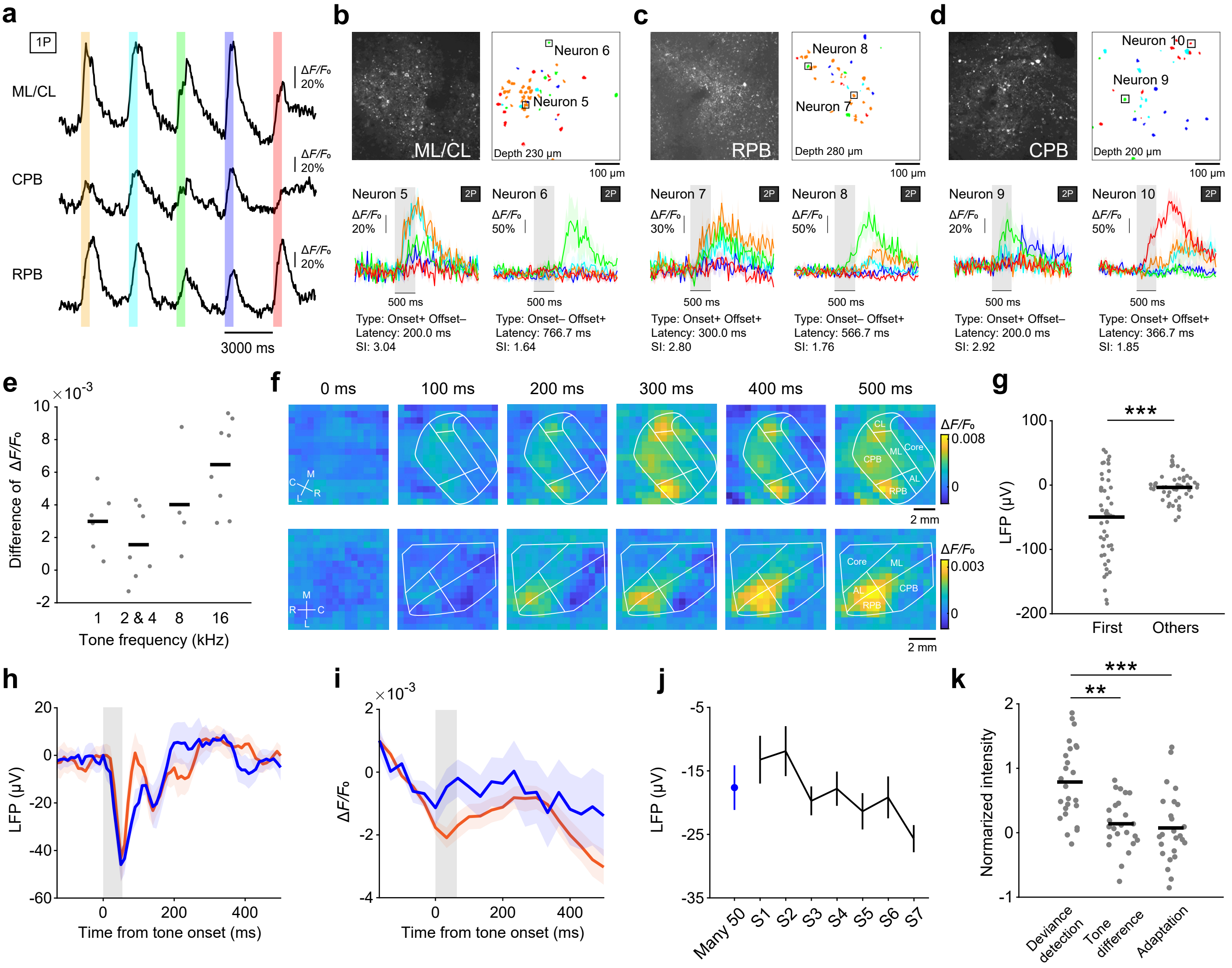

a
1P
ML/CL
CPB
RPB
ΔF/F₀ 20%
3000 ms
b
ML/CL
Neuron 6
Neuron 5
Depth 230 μm
100 μm
Neuron 5 2P
Neuron 6 2P
500 ms
Type: Onset+ Offset–
Latency: 200.0 ms
SI: 3.04
Type: Onset– Offset+
Latency: 766.7 ms
SI: 1.64
c
RPB
Neuron 8
Neuron 7
Depth 280 μm
Neuron 7 2P
Neuron 8 2P
Type: Onset+ Offset+
Latency: 300.0 ms
SI: 2.80
Type: Onset– Offset+
Latency: 566.7 ms
SI: 1.76
d
CPB
Neuron 10
Neuron 9
Depth 200 μm
Neuron 9 2P
Neuron 10 2P
Type: Onset+ Offset–
Latency: 200.0 ms
SI: 2.92
Type: Onset+ Offset+
Latency: 366.7 ms
SI: 1.85
e
Difference of ΔF/F₀
Tone frequency (kHz)
1
2 & 4
8
16
f
0 ms
100 ms
200 ms
300 ms
400 ms
500 ms
CL
ML
Core
CPB
AL
RPB
2 mm
g
LFP (μV)
First
Others
***
h
LFP (μV)
Time from tone onset (ms)
i
ΔF/F₀
Time from tone onset (ms)
j
LFP (μV)
Many 50
S1
S2
S3
S4
S5
S6
S7
k
Normarized intensity
Deviance detection
Tone difference
Adaptation
**
***

Supplementary Fig. 3. RPB shows strong deviance detection irrespective of the tone frequency used for the dMMN paradigm. **a**, Example calcium traces from parts of ML/CL, CPB, and RPB shown in Fig. 3a. These were acquired by one-photon imaging. Each color bar indicates the presentation timing of the corresponding tone. **b**–**d**, Top left, two-photon images of the same parts of ML/CL (**b**), RPB (**c**), and CPB (**d**) as in **a**. Neurons 6, 8, and 10 showed large offset responses. The response types, latencies (time to exceed half of the peak amplitude), and selectivity index (SI) are listed below each figure. Shading on each line indicates SEM. **e**, The mean amplitude of the deviance detection in each tone frequency that was used for the oddball paradigm (averaged over 200–400 ms after the tone onset) of one-photon responses in the RPB. There was no significant difference among the four groups (Black bars indicate the mean. $P > 0.05$, Wilcoxon rank sum test with Bonferroni correction, two-sided, $n = 25$; 6 for 1 kHz, 5 for 2 kHz, 2 for 4 kHz, 4 for 8 kHz, and 8 for 16 kHz, from two animals). Seven sessions at 2 kHz and 4 kHz were pooled. **f**, Paradigm-averaged responses in the field of view shown in Fig. 3a (top) and Fig. 3c (bottom) to the corresponding deviance detection. The field of view size was down-sampled to 16 × 16 pixels. The data were averaged over four dMMN paradigms for 1 kHz, one for 2 kHz, two for 8 kHz, and three for 16 kHz (top), and averaged over two dMMN paradigms for 1 kHz, four for 2 kHz, two for 4 kHz, two for 8 kHz, and five for 16 kHz (bottom). **g**, Averaged amplitude of LFP (during 0–200 ms after the tone onset) from the RPB in the many-standards control paradigm when the duration of the first tone was 100 ms. Left is the response to the first 100-ms tone and right is the average response to the 100-ms tones during the paradigm. Black bars indicate the mean. *** $P = 4.9 \times 10^{-5}$, $d = 0.97$, Wilcoxon signed-rank test, two-sided. $n = 50$ sessions from one animal, one of those used in Fig. 3h; $n = 18$ at 1 kHz, 17 at 2 kHz, and 15 at 4 kHz. **h**, Averaged LFP responses from the RPB to the 50-ms tone in the oddball paradigm (orange) and the 50-ms tone in the many-standards control paradigm (blue) ($n = 9$ penetrations as the same as Fig. 3g). In the former responses, only the tone stimulus immediately before the deviant tone stimulus was chosen in the oddball paradigm. There was not any timepoint at which the response amplitude was significantly different ($P > 0.05$, Wilcoxon rank sum test, two-sided, FDR-adjusted). The averaged amplitude during 200–400 ms after the tone onset was also not significantly different ($P = 0.37$, paired *t*-test, two-sided). Shading on each line indicates SEM. **i**, Averaged time course of calcium responses from the RPB to the 50-ms tone in the oddball paradigm (orange) and the 50-ms tone in the many-standards control paradigm (blue). In the former responses, only the tone stimulus immediately before the deviant tone stimulus was chosen ($n = 25$ dMMN paradigms as the same as Fig. 3f). There was not any timepoint at which the response amplitude was significantly different ($P > 0.05$, Wilcoxon rank sum test, two-sided, FDR-adjusted). The averaged amplitude during 200–400 ms after the tone onset was also not significantly different ($P = 0.58$, paired *t*-test, two-sided). Shading on each line indicates SEM. **j**, The left-most blue dot shows the averaged LFP amplitude to the 50-ms tone in the many-standards control paradigm in the RPB over 0–200 ms after the tone onset ($n = 9$ penetrations from one animal that was used in Fig. 3g). The other plots show the averaged LFP amplitudes in the RPB to the *i*th 50-ms tone (labeled S*i*, $i = 1$–7) after the deviant tone stimulus in the oddball paradigm ($n = 9$ penetrations from the same animal). Vertical lines indicate SEMs. The baseline amplitude was subtracted. **k**, Contributions of the three components in the RPB. The data are the same as those in Fig. 3f (RPB, $n = 25$ sessions from two animals). For each session, each component amplitude (for 200–400 ms after the tone onset) was normalized to the dMMN amplitude. Paired *t*-test with Bonferroni correction, two-sided. ** $P = 0.0013$, $d = 1.29$ for Deviance detection vs. Tone difference, *** $P = 6.9 \times 10^{-4}$, $d = 1.25$ for Deviance detection vs. Adaptation.

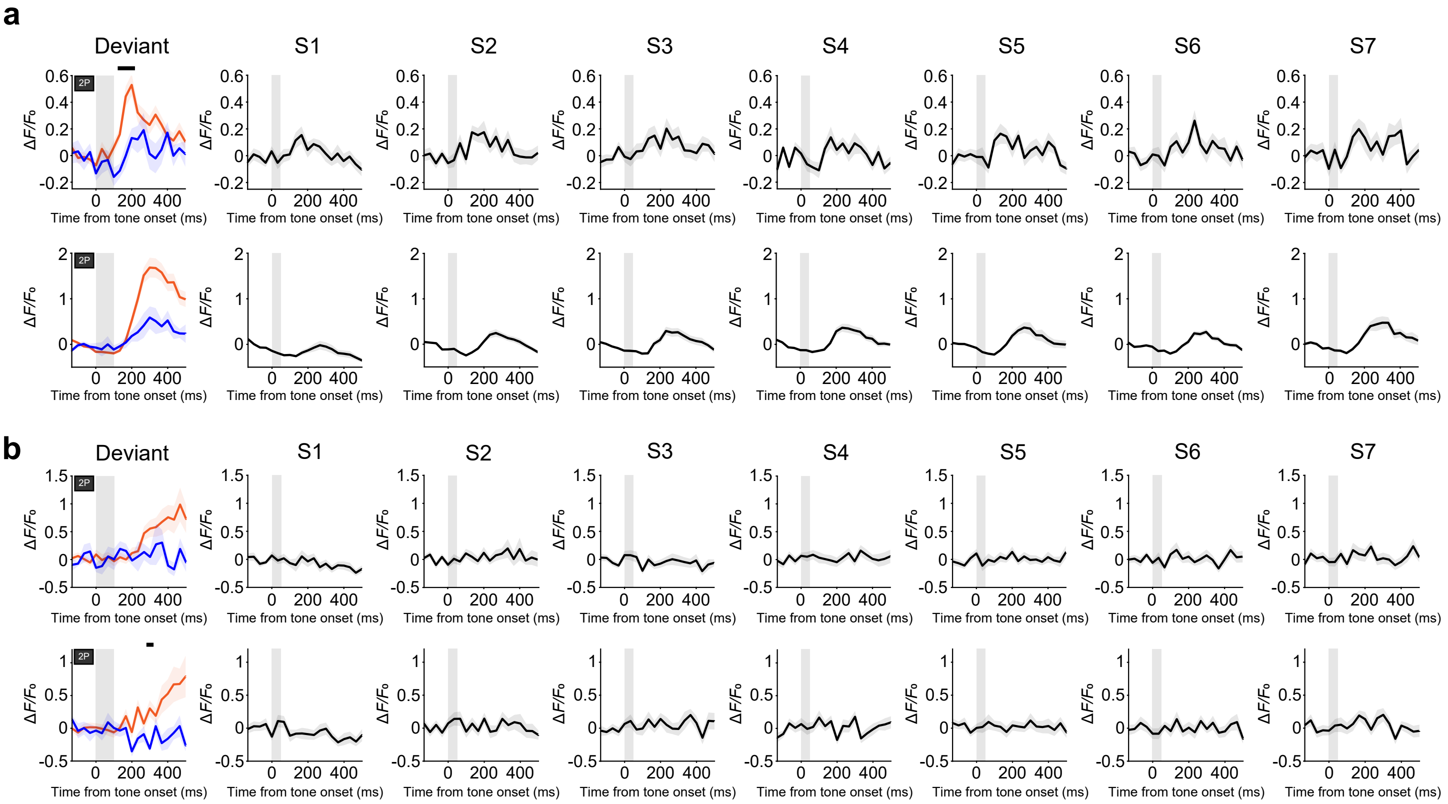

Supplementary Fig. 4. Calcium responses of L2/3 neurons during the dMMN paradigm. a,**b**, Time course of calcium responses of two representative L2/3 neurons in the core (**a**) and RPB (**b**). The left-most graphs are the responses to the deviant tone in the oddball paradigm (orange, n = 30 trials for top and 29 trials for bottom in **a**, and n = 29 trials for top and 29 trials for bottom in **b**), and the responses to the 100-ms tone in the many-standards control paradigm (blue, n = 10 trials for top and 10 trials for bottom in **a**, and n = 10 trials for top and 12 trials for bottom in **b**). Shading indicates the SEM. The black line indicates the timepoint at which the difference was significant ($P < 0.05$, Wilcoxon rank sum test, two-sided, FDR-adjusted). The p-values of the deviance detection amplitude averaged over 200–400 ms after the tone onset were $P = 0.021$, $d = 0.89$ [**a**, top], $P = 0.038$, $d = 1.04$ [**a**, bottom], $P = 0.098$, $d = 0.62$ [**b**, top], and $P = 0.0079$, $d = 0.96$ [**b**, bottom]. The responses to the ith 50-ms tone (labeled by Si) after the deviant tone stimulus in the oddball paradigm are shown from the second to eighth columns. These RPB neurons did not respond to the 100-ms stimulus in the many-standards control paradigm, in contrast to the core neurons.

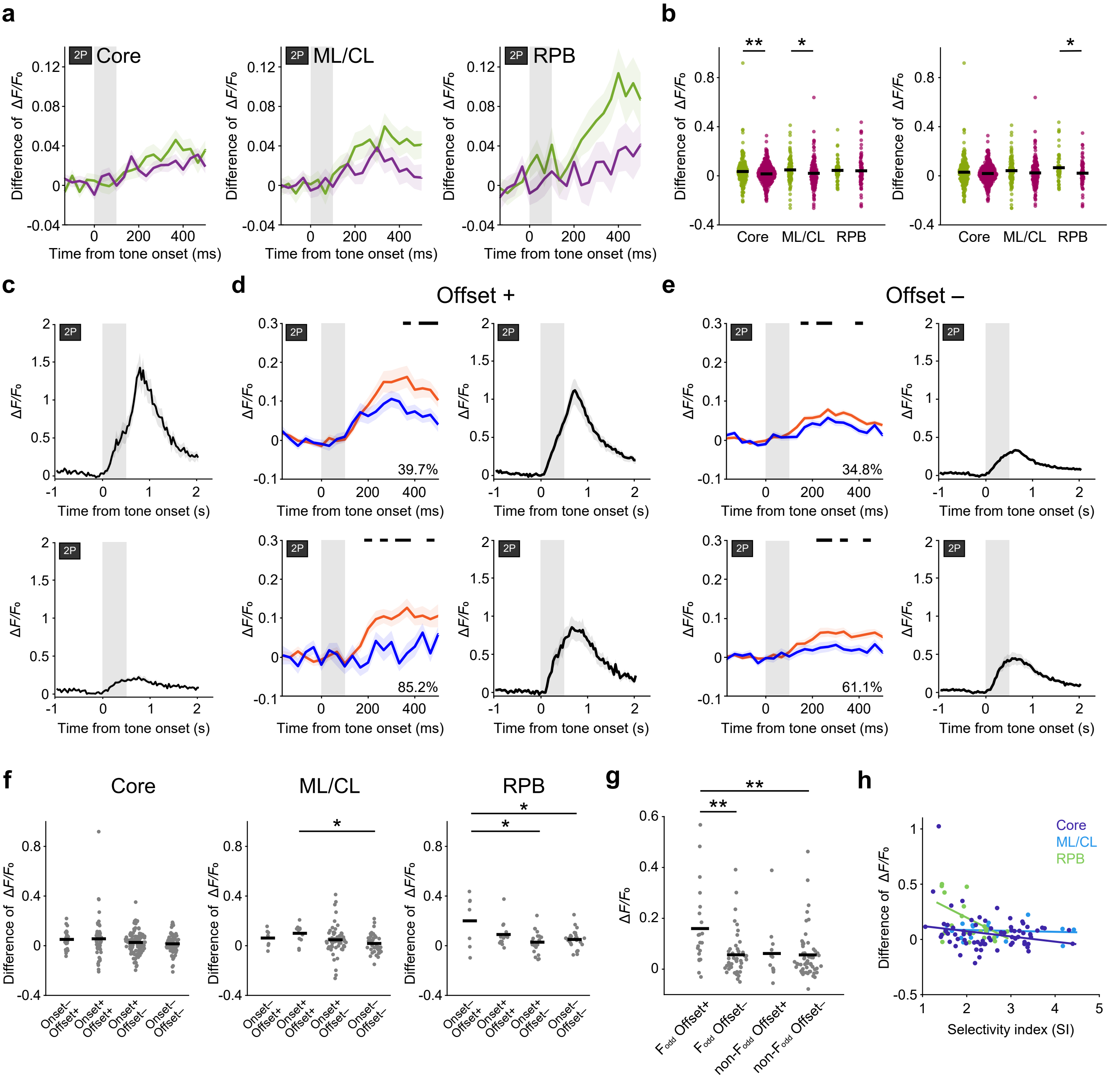
a
2P Core
2P ML/CL
2P RPB
Difference of ΔF/F₀
Time from tone onset (ms)
b
Core
ML/CL
RPB
c
d
Offset +
e
Offset –
ΔF/F₀
Time from tone onset (s)
39.7%
85.2%
34.8%
61.1%
f
Core
ML/CL
RPB
Onset– Offset+
Onset+ Offset+
Onset+ Offset–
Onset– Offset–
g
F odd Offset+
F odd Offset–
non-F odd Offset+
non-F odd Offset–
h
Core
ML/CL
RPB
Selectivity index (SI)

Supplementary Fig. 5. Deviance detection of those neurons whose BF was F_{odd} and those whose BF was otherwise. a, Time course of deviance detection in the dMMN-responsive neurons from core, ML/CL, and RPB. Green, the average deviance detection in the neurons whose BF was F_{odd} (*n* = 251 in core, 112 in ML/CL, and 68 in RPB). Purple, the average deviance detection in the neurons whose BF was not F_{odd} (*n* = 381 in core, 174 in ML/CL, and 68 in RPB). Shading on each line indicates SEM. **b**, Deviance detection amplitude (average from 200 ms after the tone onset to 400 ms after the onset) of dMMN-responsive neurons whose BF was F_{odd} (green) or not (purple) in each region (unpaired test, two-sided) when BF was determined from the response averaged over 500 ms after the tone onset (left) or after the tone end (right) in the pure-tone paradigm. These results suggest that the neurons with strong onset responses to the F_{odd} tone greatly contributed to deviance detection in the core and belt, whereas the neurons with strong offset responses to F_{odd} tone greatly did so in the RPB (** $P = 0.0081$, $d = 0.22$ [core] and * $P = 0.034$, 0.26 [ML/CL] in left, * $P = 0.021$, $d = 0.40$ [RPB] in right). **c**, Time course of calcium responses of the RPB neurons shown in Fig. 5b (upper) and Fig. 5c (lower) to their BF tone in the pure-tone paradigm. Shading on each line indicates SEM. **d**,**e**, Left, time course of calcium responses of core (upper) and ML/CL (lower) neurons whose BF was F_{odd} with offset responses (**d**), and without offset responses (**e**), to the deviant stimulus (orange) and the control stimulus (blue). dMMN-responsive neurons whose BF was F_{odd} were pooled (core, *n* = 71 [offset+], *n* = 180 [offset–]; ML/CL, *n* = 20 [offset+], *n* = 92 [offset–]). Shading indicates the SEM. The proportion of the deviance detection amplitude to the amplitude of responses to the deviant tone in the oddball paradigm is also shown. Right, time course of calcium responses of these neurons to their BF tone in the pure-tone paradigm. The black line indicates the timepoint at which the difference was significant ($P < 0.05$, Wilcoxon rank sum test, two-sided, FDR-adjusted). **f**, Deviance detection amplitude (averaged from 200–400 ms after the tone onset) in each response timing criterion. Black bars indicate the mean. Unpaired *t*-test with Bonferroni correction, two-sided, * $P = 0.012$, $d = 1.05$ (ML/CL), * $P = 0.036$, $d = 1.32$ and * $P = 0.021$, $d = 1.34$ (RPB). Core, *n* = 22 (onset–, offset+), *n* = 49 (onset+, offset+), *n* = 116 (onset+, offset–), *n* = 64 (onset–, offset–). ML/CL, *n* = 7 (onset–, offset+), *n* = 13 (onset+, offset+), *n* = 55 (onset+, offset–), *n* = 37 (onset–, offset–). RPB, *n* = 7 (onset–, offset+), 14 (onset+, offset+), 20 (onset+, offset–), and 27 (onset–, offset–). **g**, Response amplitude (averaged from 333 to 533 ms after the tone onset) of four types of RPB neurons to the 225-ms tone in the many-standards control paradigm. The neurons with and without offset responses were further divided into those whose BF was F_{odd} (F_{odd} Offset+ and F_{odd} Offset–) and those whose BF was not F_{odd} (non-F_{odd} Offset+ and non-F_{odd} Offset–). Black bars indicate the mean. ** $P < 0.01$, unpaired *t*-test with Bonferroni correction, two-sided. ** $P = 0.0050$, $d = 0.92$ for F_{odd} Offset+ vs. F_{odd} Offset–, ** $P = 0.0040$, $d = 0.91$ for F_{odd} Offset+ vs. non-F_{odd} Offset–, *n* = 21 for F_{odd} Offset+, 47 for F_{odd} Offset–, 12 for non-F_{odd} Offset+, and 56 for non-F_{odd} Offset– from two animals. There was no significant difference between F_{odd} vs. non-F_{odd} (0.088 ± 0.015 vs. 0.057 ± 0.012, *n* = 68, $P = 0.099$, unpaired *t*-test, two-sided, $d = 0.28$). **h**, Relationship between the mean amplitude of the deviance detection (averaged over 200–400 ms after the tone onset) and SI in offset+ neurons. Pearson correlation coefficients: core, $r = –0.23$, $P = 0.06$ (dark blue); ML/CL, $r = –0.10$, $P = 0.67$ (light blue); RPB, $r = –0.54$, $P = 0.011$ (green).

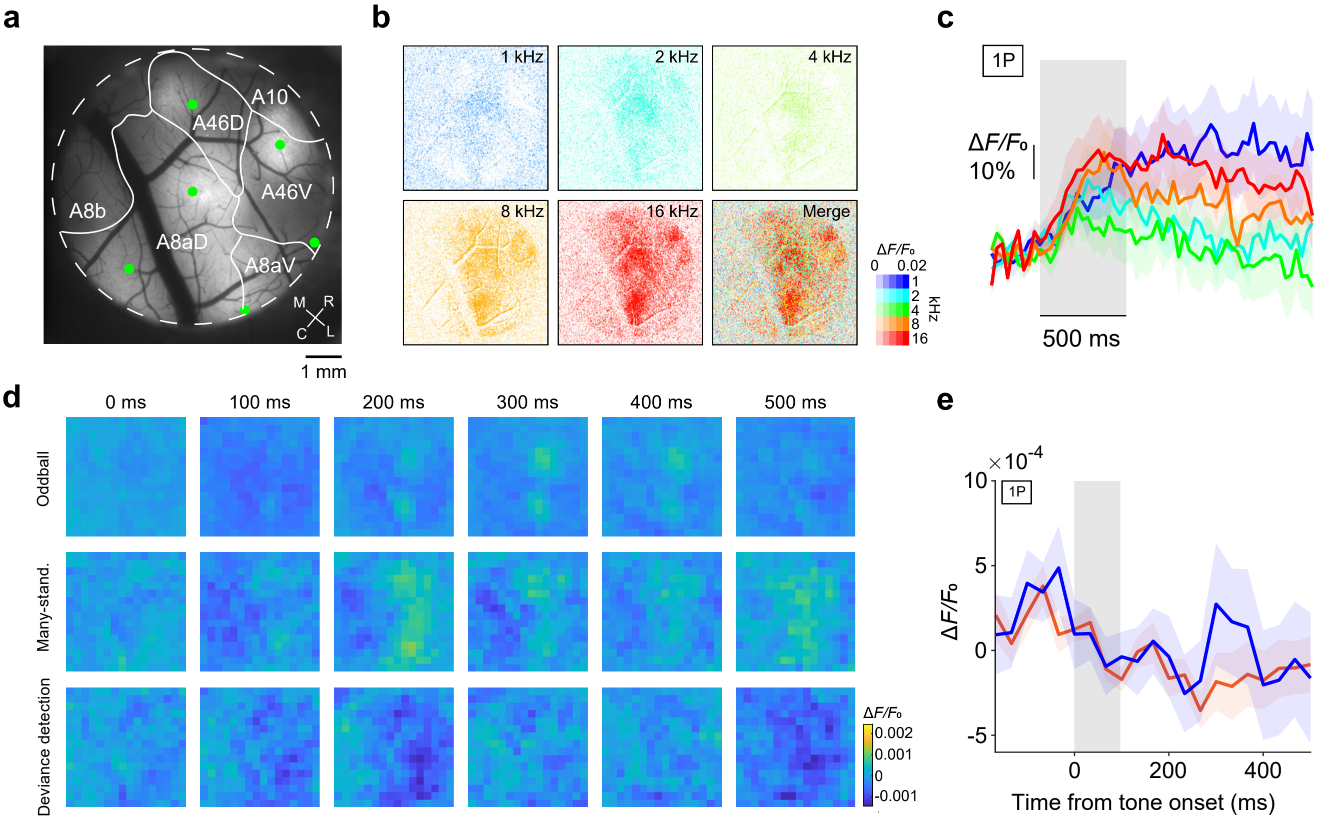

Supplementary Fig. 6. Deviance detection is very weak in the dorsolateral PFC. a, Epi-fluorescence image of the left dorsolateral PFC. Green dots indicate the AAV-injection sites. The broken white circumference indicates the outer line of an imaging window. The solid white lines indicate the putative contours of the sub-regions in areas 8, 10, and 46. Similar images were obtained in the total 23 sessions from one animal. **b**, Calcium response maps for five pure tones (1, 2, 4, 8, and 16 kHz). Bottom right, the best frequency map. For each pixel of the 256 × 6 pixel region, the color of the corresponding BF and amplitude was assigned. **c**, Averaged time course of the tone responses in the imaging window (n = 11 sessions). Shading on each line indicates SEM. **d**, Averaged map of calcium responses to the deviant tone in the field of view shown in **a**, in the oddball (top) and many-standards control (middle) paradigms, and averaged corresponding deviance detection map (bottom; n = 23: 5 for 1 kHz, 3 for 2 kHz, 5 for 4 kHz, 5 for 8 kHz, and 5 for 16 kHz). The size was down-sampled to 16 × 16 pixels. **e**, Session-averaged time courses of calcium responses to the 100-ms tone in the oddball (orange) and many-standards control (blue) paradigms (23 maps). There was not any timepoint at which the response amplitude was significantly different ($P > 0.05$, Wilcoxon rank sum test, two-sided, FDR-adjusted). Shading on each line indicates SEM.

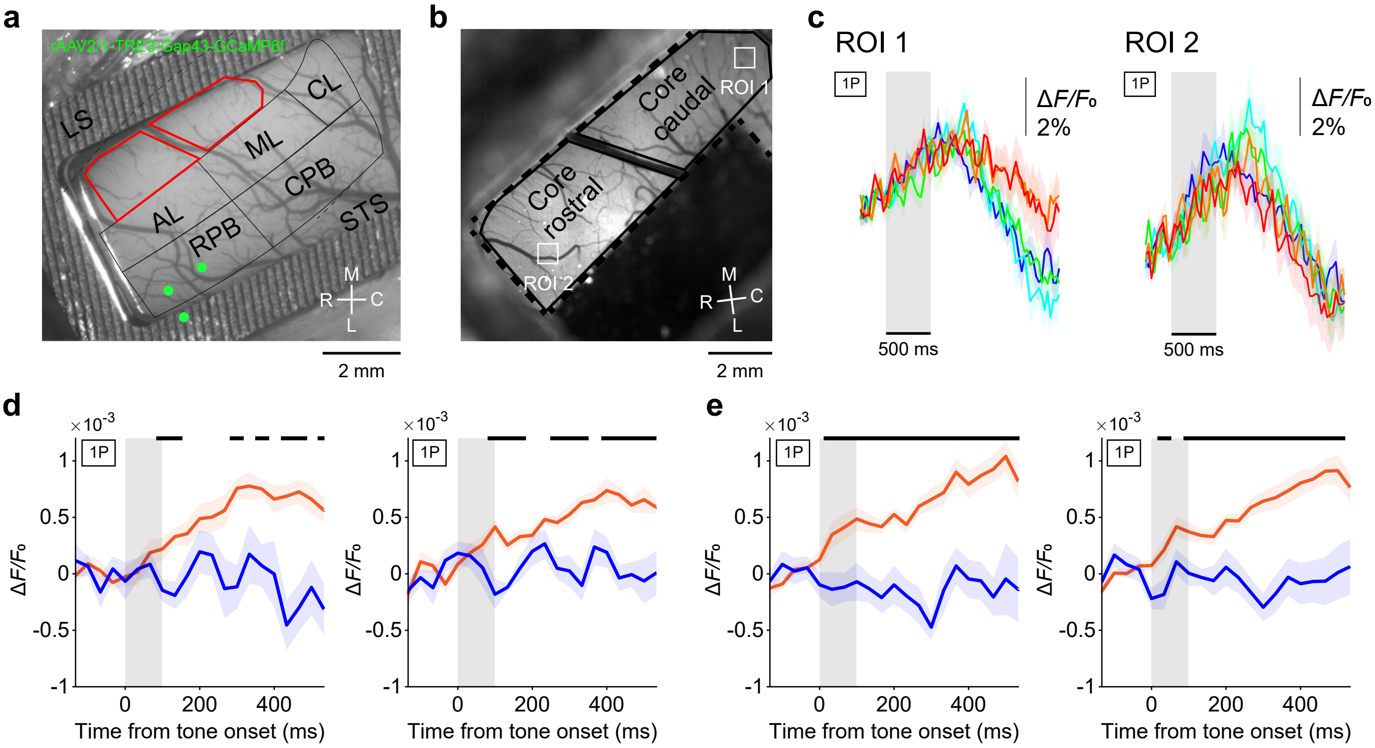

Supplementary Fig. 7. Deviance detection signal from RPB spreads throughout the core irrespective of tone frequency. **a**, Vasculature image of the left auditory cortex. Green dots indicate the GAP43-GCaMP6f injection sites. The solid black lines indicate the putative contours of the sub-regions of the auditory cortex. Two red contours correspond to the rostral and caudal parts of the core shown in Fig. 6b. Similar images were obtained in the total 27 sessions from one animal. **b**, Epi-fluorescence image of the left auditory cortical areas that expressed GAP43-GCaMP6f. Broken lines indicate the outer lines of the imaging window and solid lines indicate the contours of the core that was inferred from an atlas. To avoid fluorescence emission from the vicinity of the RPB injection site, a black sheet was placed on the cover glass above the lateral belt and parabelt. **c**, Representative time courses of calcium responses to five pure tones from two ROIs in **b**. Shading on each line indicates SEM. **d**, Session-averaged time courses of calcium responses to the 100-ms tone in the oddball (orange) and many-standards control (blue) paradigms in the deviant tone responsive regions in the rostral (left) and caudal (right) parts of the core when F_{odd} was 1 and 2 kHz. Left, n = 9 sessions from one animal. Right; n = 8 sessions from one animal. Shading indicates the SEM. The black line indicates the timepoint at which the difference was significant ($P < 0.05$, Wilcoxon rank sum test, two-sided, FDR-adjusted). **e**, Session-averaged time courses of calcium responses to the 100-ms tone in the oddball (orange) and many-standards control (blue) paradigms in the deviant tone responsive regions in the rostral (left) and caudal (right) parts of the core when F_{odd} was 8 and 16 kHz. Left, n = 13 sessions from one animal. Right; n = 13 sessions from one animal. The black line indicates the timepoint at which the difference was significant ($P < 0.05$, Wilcoxon rank sum test, two-sided, FDR-adjusted). Shading on each line indicates SEM.

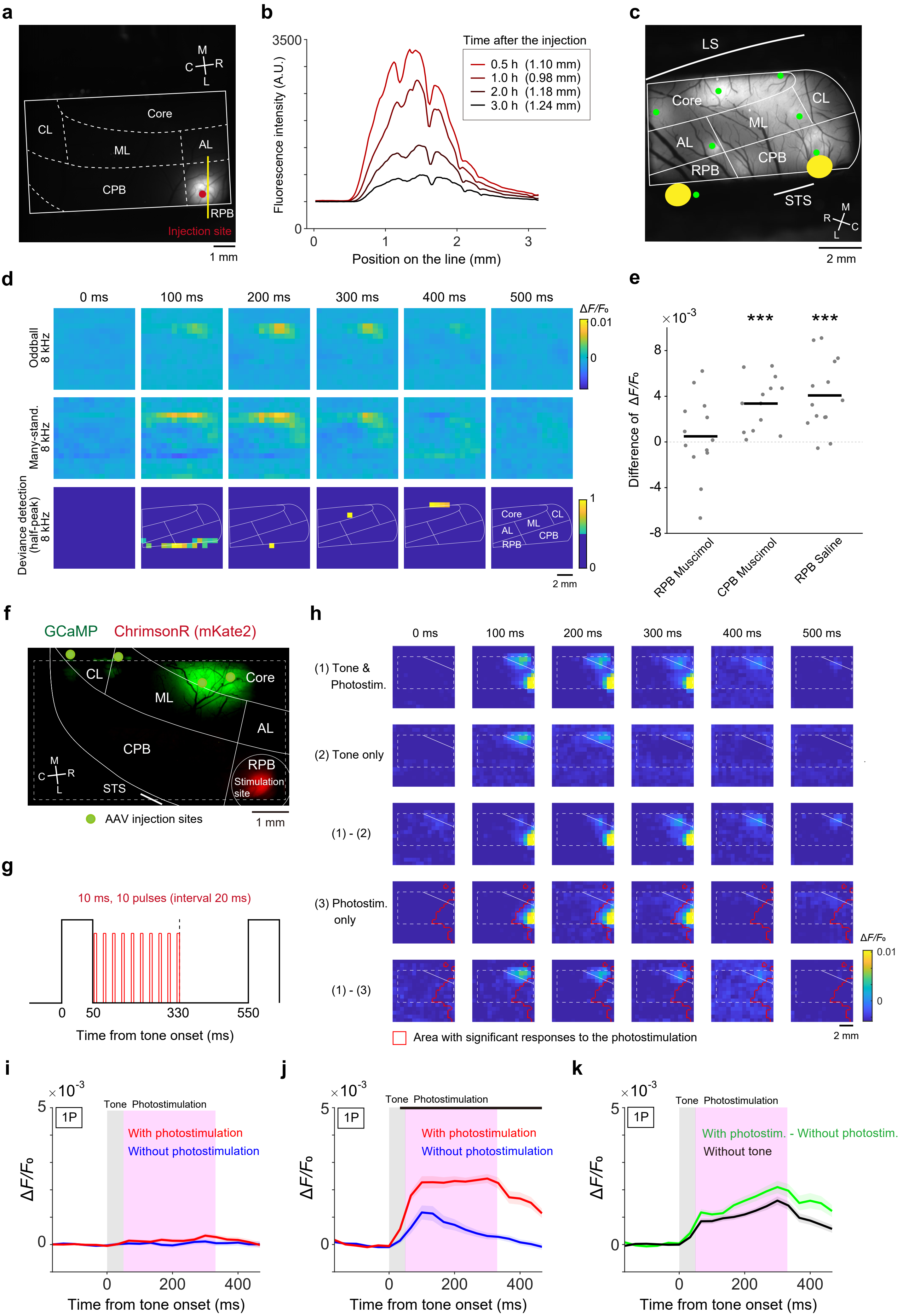

a
M
C
R
L
Core
CL
ML
AL
CPB
RPB
Injection site
1 mm
b
3500
0
Fluorescence intensity (A.U.)
Time after the injection
0.5 h (1.10 mm)
1.0 h (0.98 mm)
2.0 h (1.18 mm)
3.0 h (1.24 mm)
0
1
2
3
Position on the line (mm)
c
LS
Core
CL
ML
AL
CPB
RPB
STS
M
R
C
L
2 mm
d
0 ms
100 ms
200 ms
300 ms
400 ms
500 ms
ΔF/F₀
0.01
0
Oddball 8 kHz
Many-stand. 8 kHz
Deviance detection (half-peak) 8 kHz
1
0
Core
CL
ML
AL
CPB
RPB
2 mm
e
×10-3
***
***
8
4
0
-4
-8
Difference of ΔF/F₀
RPB Muscimol
CPB Muscimol
RPB Saline
f
GCaMP
ChrimsonR (mKate2)
CL
Core
ML
AL
CPB
RPB
Stimulation site
STS
M
C
R
L
AAV injection sites
1 mm
g
10 ms, 10 pulses (interval 20 ms)
0
50
330
550
Time from tone onset (ms)
h
0 ms
100 ms
200 ms
300 ms
400 ms
500 ms
(1) Tone & Photostim.
(2) Tone only
(1) - (2)
(3) Photostim. only
(1) - (3)
ΔF/F₀
0.01
0
Area with significant responses to the photostimulation
2 mm
i
×10-3
5
0
1P
Tone
Photostimulation
With photostimulation
Without photostimulation
ΔF/F₀
0
200
400
Time from tone onset (ms)
j
×10-3
5
0
1P
Tone
Photostimulation
With photostimulation
Without photostimulation
ΔF/F₀
0
200
400
Time from tone onset (ms)
k
×10-3
5
0
1P
Tone
Photostimulation
With photostim. - Without photostim.
Without tone
ΔF/F₀
0
200
400
Time from tone onset (ms)

Supplementary Fig. 8. Experiments to examine the effects of RPB on the core responses. a,**b**, Fluorescence of BODIPY-conjugated muscimol solution injected into RPB. **a**, Red dot indicates the injection site. The example image was acquired 2 hours after the injection. **b**, Fluorescence profiles along the line crossing near the injection center (yellow line in **a**). The full widths at half maximum of the intensity profiles at each timepoint are indicated in parentheses. **c**, Top, epi-fluorescence image of the left auditory cortical areas that expressed GCaMP6f. Yellow spots indicate muscimol-injection sites. Similar images were obtained in the total 40 sessions from two animals. **d**, Representative trial-averaged response map of the core and belt in oddball (top) and many-standards (middle) paradigms with an 8-kHz tone (upper and middle row) and corresponding deviance detection (bottom) after muscimol injection into RPB, as shown in **c**. The pixel size was down-sampled to 16 × 16 pixels. In the deviance detection map, the pixels with less than half of the maximum response are set to the same deep blue color. White lines indicate the outer lines of an imaging window or putative contours of the sub-regions of the auditory cortex. **e**, Early deviance detection amplitude (averaged over 100–167 ms after the tone onset) in each condition (Fig. 7a–c). One-sample *t*-test, two-sided. *** $P = 1.9 \times 10^{-4}$ (CPB Muscimol), *** $P = 3.0 \times 10^{-4}$ (RPB Saline). RPB muscimol, $n = 13$ sessions; CPB muscimol, $n = 13$ sessions; RPB saline, $n = 14$ sessions. **f**, Epi-fluorescence image of the right auditory cortical areas that expressed GCaMP6f in the core and ChrimsonR-mKate2 in the RPB. Green dots indicate the injection sites of AAV carrying GCaMP6f. Another injection site in the core was outside the glass window. The green and red fluorescence with intensity above the mean plus two standard deviations of the intensity in the blood vessel are overlaid. Similar images were obtained in the total 68 sessions from three animals. **g**, Time sequence of the tone stimulation and RPB photostimulation. **h**, Representative trial-averaged response maps with tone presentation and photostimulation (top row, 1); trial-averaged response maps with only tone presentation (second row from top, 2); maps of the top row minus the maps of the second row (third row, 1–2); representative trial-averaged response maps with only photostimulation (fourth row, 3); and maps of the top row minus the maps of the fourth row (bottom, 1–3). The pixel size wa s down-sampled to 16 × 16 pixels. Red contours indicate the areas that showed a significant response to only photostimulation on the same day and solid lines indicate the border between core and belt areas. The core area included in these areas was excluded from the calculation of the core response as in Fig. 7f,g. **i**, Calcium responses in the core area to photostimulation of RPB (red) and to no photostimulation (blue). The dummy tone presentation period was set in both conditions. The core area was determined in the same way as in Fig. 7f. There was no timepoint at which the difference was significant ($P > 0.05$, Wilcoxon rank sum test, two-sided, FDR-adjusted). $n = 68$ from three animals; 19 for 1 kHz, 16 for 2 kHz, 15 for 4 kHz, 13 for 8 kHz, and 5 for 16 kHz from three animals. Shading on each line indicates SEM. **j**, Calcium responses to the tone followed by photostimulation (red) and to only the tone (blue) in the core region that showed significant responses to the tone followed by photostimulation and to only photostimulation. The black horizontal line indicates the period during which the amplitude significantly differed between the red and blue traces ($P < 0.05$, Wilcoxon rank sum test, two-sided, FDR-adjusted). $n = 46$ from three animals; 14 for 1 kHz, 11 for 2 kHz, 11 for 4 kHz, 9 for 8 kHz, and 1 for 16 kHz. The reason why this session number (46) was smaller than that in Fig. 7f (68) was because no core region showed significant responses to both the tone followed by the photostimulation and to only the photostimulation in 22 sessions. Shading on each line indicates SEM. **k**, Black, calcium responses to only photostimulation in the core region that showed significant responses to the tone followed by the photostimulation and to only the photostimulation. Green, the red response minus the blue response in **j**. There was no timepoint at which the black and green traces significantly differed ($P > 0.05$, Wilcoxon rank sum test, two-sided, FDR-adjusted). For the black response, $n = 54$ from three animals. The reason why the session number differed between the black and green responses was because the number of sessions with only photostimulation and the number of sessions with the tone followed by the photostimulation were different. Shading on each line indicates SEM.

Abbreviations	Full spelling
1P	One-photon calcium imaging
2P	Two-photon calcium imaging
A1	Primary auditory cortex
AAV	Adeno-associated virus
AL	Anterolateral belt
BF	Best tone frequency
CL	Caudolateral belt
CPB	Caudal parabelt
dlPFC	Dorsolateral prefrontal cortex
dMMN	Duration mismatch negativity
FDR	False discovery rate
F_{odd}	Tone frequency used in the oddball paradigm
L1	Layer 1
L2/3	Layer 2/3
LFP	Local field potential
ML	Mediolateral belt
MMN	Mismatch negativity
ROI	Region of interest
RPB	Rostral parabelt
SI	Selectivity index for response frequency
SOA	Stimulus onset asynchrony

Supplementary Table 1. Abbreviation list

		Core	ML/CL	RPB	CPB
**Onset+ Offset– (%)**	**Num. of cells**	636 (37%)	295 (41%)	93 (28%)	94 (30%)
	**Latency**	258 ± 6 ms	274 ± 9 ms	219 ± 14 ms	263 ± 21 ms
**Onset+ Offset+ (%)**	**Num. of cells**	227 (13%)	60 (8%)	42 (13%)	23 (7%)
	**Latency**	376 ± 10 ms	380 ± 20 ms	398 ± 23 ms	357 ± 36 ms
**Onset– Offset+ (%)**	**Num. of cells**	162 (10%)	58 (8%)	31 (9%)	23 (7%)
	**Latency**	461 ± 17 ms)	452 ± 28 ms	453 ± 46 ms	503 ± 44 ms
**Onset– Offset– (%)**	**Num. of cells**	678 (40%)	312 (43%)	169 (50%)	178 (56%)
	**Latency**	255 ± 7 ms	288 ± 11 ms	266 ± 16 ms	305 ± 17 ms
**Total number**		1703	725	335	318
**Animal number**		3	2	2	2
**Imaging field number**		34	16	11	11
**SI (Z-scored)**		3.41 ± 0.09	3.42 ± 0.14	3.60 ± 0.10	3.55 ± 0.21
**BF**	**1 kHz**	198 (12%)	171 (23%)	56 (16%)	120 (38%)
	**2 kHz**	525 (31%)	63 (9%)	29 (9%)	46 (15%)
	**4 kHz**	411 (24%)	109 (15%)	36 (11%)	64 (20%)
	**8 kHz**	294 (17%)	145 (20%)	151 (45%)	20 (6%)
	**16 kHz**	275 (16%)	237 (33%)	63 (19%)	68 (21%)

Supplementary Table 2. Pure-tone response properties in core, ML/CL, RPB, and CPB neurons. Number, proportion, and latency (mean ± SEM) of onset+ offset– neurons, onset+ offset+ neurons, onset–offset+ neurons, and onset–offset– neurons, and the selectivity index (SI) and best tone frequency (BF) of individual tone-responsive neurons in the core, ML/CL, RPB, and CPB. SI (range, 5–1) is an indicator of the sharpness of the frequency tuning (see Methods). Latency was defined as the time from the tone onset to the point when the response exceeded half of the maximal amplitude in the pure-tone paradigm.
