# Supplementary material for: Change detection in the primate auditory cortex through feedback of prediction error signals
Source: Nat Commun. 2023 Nov 13;14:6981. doi: 10.1038/s41467-023-42553-3 (PMC10643402; doi:10.1038/s41467-023-42553-3)
Supplement: Supplementary file 3 — Reporting Summary [file 41467_2023_42553_MOESM3_ESM.pdf]

nature portfolio

Corresponding author(s): Masanori Matsuzaki

Last updated by author(s): Oct 3, 2023

Reporting Summary

Nature Portfolio wishes to improve the reproducibility of the work that we publish. This form provides structure for consistency and transparency in reporting. For further information on Nature Portfolio policies, see our Editorial Policies and the Editorial Policy Checklist.

Statistics

For all statistical analyses, confirm that the following items are present in the figure legend, table legend, main text, or Methods section.

n/a	Confirmed	
☐	☒	The exact sample size (*n*) for each experimental group/condition, given as a discrete number and unit of measurement
☐	☒	A statement on whether measurements were taken from distinct samples or whether the same sample was measured repeatedly
☐	☒	The statistical test(s) used AND whether they are one- or two-sided<br>*Only common tests should be described solely by name; describe more complex techniques in the Methods section.*
☐	☒	A description of all covariates tested
☐	☒	A description of any assumptions or corrections, such as tests of normality and adjustment for multiple comparisons
☐	☒	A full description of the statistical parameters including central tendency (e.g. means) or other basic estimates (e.g. regression coefficient) AND variation (e.g. standard deviation) or associated estimates of uncertainty (e.g. confidence intervals)
☐	☒	For null hypothesis testing, the test statistic (e.g. *F*, *t*, *r*) with confidence intervals, effect sizes, degrees of freedom and *P* value noted<br>*Give P values as exact values whenever suitable.*
☒	☐	For Bayesian analysis, information on the choice of priors and Markov chain Monte Carlo settings
☒	☐	For hierarchical and complex designs, identification of the appropriate level for tests and full reporting of outcomes
☒	☐	Estimates of effect sizes (e.g. Cohen's *d*, Pearson's *r*), indicating how they were calculated

Our web collection on statistics for biologists contains articles on many of the points above.

Software and code

Policy information about availability of computer code

Data collection	One-photon imaging was conducted with a variable zoom microscope (Axio Zoom.V16; Carl Zeiss, Jena, Germany) with a 2.3× objective (Plan-NEOFLUAR Z 2.3×/0.57; Carl Zeiss)37. A monochromic EM-CCD camera with 1024×1024 pixels (iXon Ultra 888; Andor Technology, Belfast, UK), or an sCMOS camera with 2048 × 2048 pixels (SONA 4bv-11; Andor Technology) was used as a photodetector. Images were acquired using a 470 nm blue LED (M470L3; Thorlabs, NJ, USA). Two-photon imaging was conducted with a custom-built two-photon microscope (Olympus, Tokyo, Japan) equipped with a water immersion objective lens (XLPLN10XSVMP, numerical aperture of 0.6, working distance of 8 mm; XLSLPLN25XSVMP2, numerical aperture of 0.95, working distance of 8 mm; Olympus). Extracellular data were collected with DAM80 (World Precision Instruments) with tungsten microelectrodes (500 kΩ; World Precision Instruments).
Data analysis	All calcium imaging data were analyzed using MATLAB (R2018a, 9.4.0.949201; MathWorks, MA, USA), ImageJ (National Institute of Health, Bethesda, MD, USA). Turboreg (http://bigwww.epfl.ch/thevenaz/turboreg/), NoRMCorre (https://github.com/flatironinstitute/NoRMCorre), and CaImAn (https://github.com/flatironinstitute/CaImAn-MATLAB) were used for the analysis. All extracellular recording data were analyzed using MATLAB(R2018a, 9.4.0.949201; MathWorks, MA, USA).

For manuscripts utilizing custom algorithms or software that are central to the research but not yet described in published literature, software must be made available to editors and reviewers. We strongly encourage code deposition in a community repository (e.g. GitHub). See the Nature Portfolio guidelines for submitting code & software for further information.

Data

Policy information about availability of data

All manuscripts must include a data availability statement. This statement should provide the following information, where applicable:

- Accession codes, unique identifiers, or web links for publicly available datasets
- A description of any restrictions on data availability
- For clinical datasets or third party data, please ensure that the statement adheres to our policy

All data and computer codes are available from the corresponding author.

Research involving human participants, their data, or biological material

Policy information about studies with human participants or human data. See also policy information about sex, gender (identity/presentation), and sexual orientation and race, ethnicity and racism.

Reporting on sex and gender	*Use the terms sex (biological attribute) and gender (shaped by social and cultural circumstances) carefully in order to avoid confusing both terms. Indicate if findings apply to only one sex or gender; describe whether sex and gender were considered in study design; whether sex and/or gender was determined based on self-reporting or assigned and methods used. Provide in the source data disaggregated sex and gender data, where this information has been collected, and if consent has been obtained for sharing of individual-level data; provide overall numbers in this Reporting Summary. Please state if this information has not been collected. Report sex- and gender-based analyses where performed, justify reasons for lack of sex- and gender-based analysis.*
Reporting on race, ethnicity, or other socially relevant groupings	*Please specify the socially constructed or socially relevant categorization variable(s) used in your manuscript and explain why they were used. Please note that such variables should not be used as proxies for other socially constructed/relevant variables (for example, race or ethnicity should not be used as a proxy for socioeconomic status). Provide clear definitions of the relevant terms used, how they were provided (by the participants/respondents, the researchers, or third parties), and the method(s) used to classify people into the different categories (e.g. self-report, census or administrative data, social media data, etc.) Please provide details about how you controlled for confounding variables in your analyses.*
Population characteristics	*Describe the covariate-relevant population characteristics of the human research participants (e.g. age, genotypic information, past and current diagnosis and treatment categories). If you filled out the behavioural & social sciences study design questions and have nothing to add here, write "See above."*
Recruitment	*Describe how participants were recruited. Outline any potential self-selection bias or other biases that may be present and how these are likely to impact results.*
Ethics oversight	*Identify the organization(s) that approved the study protocol.*

Note that full information on the approval of the study protocol must also be provided in the manuscript.

Field-specific reporting

Please select the one below that is the best fit for your research. If you are not sure, read the appropriate sections before making your selection.

☒ Life sciences ☐ Behavioural & social sciences ☐ Ecological, evolutionary & environmental sciences

For a reference copy of the document with all sections, see nature.com/documents/nr-reporting-summary-flat.pdf

Life sciences study design

All studies must disclose on these points even when the disclosure is negative.

Sample size	We did not employ statistical methods to predetermine the sample sizes; however, sample sizes were estimated according to previous methodically-comparable laboratory experiments and are similar to those generally employed in the field.
Data exclusions	In the two-photon imaging experiment, the data for four days in which both the pure-tone paradigm and the dMMN paradigm were not recorded from the same imaging plane were excluded from the analysis.
Replication	We obtained multiple one-photon imaging data (n = 189 ROIs from 14 animals), two-photon imaging data (n = 136 ROIs for neuropil analysis from 3 animals, n = 3404 neuronal somata from 5 animals), and extracellular recoding data (n = 58 penetrations from 5 animals). Reproducibility is ensured by using a sufficient number of individuals for physiological research using primates.
Randomization	Tone stimuli in the pure-tone paradigm and dMMN paradigm (oddball paradigm and many-standards control paradigm) were randomly assigned. The experimental order was generally pure-tone paradigm and dMMN paradigm, although in some cases the order was shuffled.
Blinding	As described above, all stimuli in the pure-tone paradigm and dMMN paradigm were automatically randomly assigned.

Blinding	Drug injection was conducted using a double-blind test in which the experimenter did not know whether drug or placebo was injected.

Reporting for specific materials, systems and methods

We require information from authors about some types of materials, experimental systems and methods used in many studies. Here, indicate whether each material, system or method listed is relevant to your study. If you are not sure if a list item applies to your research, read the appropriate section before selecting a response.

Materials & experimental systems

n/a	Involved in the study
☒	☐ Antibodies
☒	☐ Eukaryotic cell lines
☒	☐ Palaeontology and archaeology
☐	☒ Animals and other organisms
☒	☐ Clinical data
☒	☐ Dual use research of concern
☒	☐ Plants

Methods

n/a	Involved in the study
☒	☐ ChIP-seq
☒	☐ Flow cytometry
☒	☐ MRI-based neuroimaging

Animals and other research organisms

Policy information about studies involving animals; ARRIVE guidelines recommended for reporting animal research, and Sex and Gender in Research

Laboratory animals	This study used Nineteen laboratory-bred common marmosets (Callithrix jacchus; twelve males and seven females, age was 1–5 years).
Wild animals	This study did not use wild animals.
Reporting on sex	This study used eleven male and seven female animals. For each experiment in this study, the number of the animals was one to five. Due to the low sample size and because the sex does not significantly affect the results in the human MMN, we did not conduct sex-based analysis. For one-photon imaging of the core, three males and two females were used. Among them, one male and two females were used for two-photon imaging. For one-photon and two-photon imaging of the belt and parabelt, one male and one female were used. For LFP recording, one male and two females were used. For muscimol inactivation of the parabelt, two males were used. For structural imaging of the axons from the RPB, one female was used. For calcium imaging of the axons from the RPB, one male was used. For one-photon imaging of the dorsolateral prefrontal cortex, one male was used. For photoactivation of the RPB, one male and two females were used. For recording of LFP adaptation, two males were used. For estimating the spread of muscimol within the auditory cortex, one female was used.
Field-collected samples	This study did not use samples collected from the field.
Ethics oversight	All experiments were approved by the Animal Experimental Committee of the University of Tokyo and the Animal Care and Use Committees of the RIKEN Center for Brain Science.

Note that full information on the approval of the study protocol must also be provided in the manuscript.
